# Seafarer fatigue: a systematic review and narrative synthesis of concepts, risk factors, prediction methods, and risk management strategies

**DOI:** 10.3389/fpubh.2026.1835115

**Published:** 2026-07-13

**Authors:** Zhiwei Zhao, Anqi Quan, Shuxin He, Jorgen Riis Jepsen, Wessel M. A. van Leeuwen

**Affiliations:** 1 College of Transportation Engineering, Dalian Maritime University, Dalian, Liaoning, China; 2 Department of Occupational and Environmental Medicine, University Hospital of Southern Denmark, Grindsted, Denmark; 3 Department of Psychology, Stockholm University, Stockholm, Sweden

**Keywords:** comparative study, fatigue, fatigue prediction, fatigue risk management, risk factors, seafarers

## Abstract

Seafarer fatigue is a major contributing factor to maritime accidents and has gained widespread attention in the shipping industry. However, existing evidence remains fragmented across different fatigue concepts, measurement approaches, risk factors, prediction models, and management strategies. This systematic review and narrative synthesis aimed to critically synthesize current evidence on seafarer fatigue, with particular attention to its conceptualization, risk factors, subgroup differences, prediction approaches, and fatigue risk management strategies. A systematic search was conducted in Web of Science, Scopus, PubMed, CNKI, and Wanfang from January 2000 to April 2026. Additional studies were identified through citation searching. After screening and eligibility assessment, 56 studies were included. Owing to substantial heterogeneity in study designs, populations, fatigue definitions, measurement tools, and outcomes, no meta-analysis was conducted; instead, findings were synthesized narratively. The review indicates that seafarer fatigue is a multidimensional phenomenon shaped by sleep and recovery conditions, work organization, shipboard environmental stressors, individual and psychosocial factors, and organizational or regulatory contexts. This review highlights the need for clearer fatigue concepts, stronger subgroup-specific evidence, externally validated prediction models, and integrated fatigue risk management systems tailored to diverse maritime working conditions.

## 1 Introduction

### 1.1 Seafarer fatigue as an occupational and maritime safety issue

Seafarer fatigue is a major occupational health and maritime safety concern. As the primary operators of vessels, seafarers are responsible for navigation, engine operation, cargo handling, emergency response, and other safety-critical tasks. Fatigue may impair vigilance, reaction time, decision-making, coordination, and situational awareness, thereby increasing the likelihood of operational errors and maritime accidents.

Fatigue adversely affects seafarers’ cognitive abilities and behavioral performance. Studies indicate that shift work and resultant fatigue can lead to judgment errors, increasing the likelihood of accidents (1). Physiologically and psychologically, fatigue is a critical factor in seafarer errors (2), causing lapses in vigilance or misjudgments that may result in grounding, collisions, or other maritime incidents (3). A number of health issues as reviewed in Jepsen et al. such as insomnia and circadian rhythm disruptions may further contribute to fatigue and to the consequences of fatigue (4).

As indicated above, the negative effects of fatigue on seafarers pose significant threats to maritime safety (5). As the primary operators of vessels, seafarers’ correct actions are vital for safe navigation, and fatigue-induced errors are a major cause of maritime accidents (6). Research indicates that human factors account for at least 60% of maritime accidents (7), with fatigue being a notable contributor. Fan found that more than 21% of maritime accidents involved reduced vigilance among crew members, while more than 16% are linked to distracted or insufficient attention (8), both of which are directly or indirectly associated with fatigue. For example, the Exxon Valdez grounding incident was partly attributed to fatigue, as the three night-shift crew members had only 5–6 h of sleep in the 24 h preceding the accident (9).

Fatigue-related maritime accidents can also have severe environmental consequences. Ships often carry large quantities of fuel and cargo, and accidents may lead to oil spills or cargo dispersal. For instance, the 2010 grounding of the coal carrier Shen Neng 1 resulted in several tons of fuel leaking into the sea, creating a 3-kilometer oil slick that severely polluted the Great Barrier Reef. Investigations revealed that the accident was caused by excessive fatigue in the officer on watch (10).

In summary, fatigue has significant negative implications for ships, the marine environment and seafarers.

Empirical data supports that fatigue is prevalent in the maritime industry. It was found that 40.6% of surveyed seafarers had fallen asleep at work at least once in the past 5 years (11). A study on Lithuanian seafarers reported that 76.3% of respondents experienced fatigue at sea (12). Similarly, Hebbar and Mukesh found that among 288 seafarers, 40% felt unhappy, 30% were stressed, and over 15% were severely fatigued (13). In a study of over 1,800 professional seafarers a quarter reported feeling fatigued or drowsy at work (14). Cui et al. highlighted the common exposure to engine noise during sleep among seafarers, and the ensuing disruption of sleep patterns, contributing to poor sleep quality and exacerbating fatigue (15). By considering multiple studies, it was concluded that fatigue is prevalent among seafarers, with an average fatigue rate of 65%, varying between 50 and 80% across maritime sectors (16). These findings underscore the severity and pervasiveness of fatigue in maritime work.

### 1.2 Shipboard working conditions and fatigue risk

The risk of fatigue among seafarers is closely related to the distinctive working and living conditions on board ships. Unlike many shore-based occupations, seafaring combines workplace and living space within the same confined environment. Seafarers often work long hours, follow rotating watchkeeping schedules, perform night work, and remain on board for extended periods. These conditions may restrict sleep opportunities, disrupt circadian rhythms, and reduce recovery from prior work.

Work organization is a central source of fatigue risk. Watchkeeping schedules, high workload, short turnaround times in port, administrative duties, emergency tasks, and insufficient manning can all increase fatigue. In some operational contexts, seafarers may have limited opportunities for uninterrupted rest, especially during port operations, bad weather, high-traffic navigation, or intensive cargo handling. Even when formal rest-hour requirements are met, actual recovery may be affected by fragmented sleep, operational interruptions, and poor sleep quality.

Shipboard environmental conditions may further intensify fatigue. Noise, vibration, heat, motion, lighting conditions, and limited accommodation space can reduce sleep quality and contribute to physical and psychological strain. For example, Cui et al. highlighted the common exposure of seafarers to engine noise during sleep, which may disrupt sleep patterns, contribute to poor sleep quality, and exacerbate fatigue (15). These environmental stressors may interact with work demands and sleep restriction, producing cumulative fatigue over time.

Fatigue risk is also shaped by organizational and safety culture. Whether fatigue is recognized, reported, normalized, or actively managed depends on company policies, manning practices, leadership, reporting culture, and the broader safety climate on board. In multicultural crews, different national, organizational, and professional cultures may influence how fatigue is perceived, communicated, and managed. Therefore, seafarer fatigue should not be understood only as an individual condition, but as a product of interactions among work organization, shipboard environment, regulatory compliance, and safety culture.

### 1.3 Conceptual complexity of seafarer fatigue

Fatigue is characterized by its complexity and multifactorial nature, leading to diverse definitions from different scientific perspectives.

Fatigue is often categorized into physical fatigue and mental fatigue (17). Physical fatigue refers to localized pain in overworked muscles, while mental fatigue is a diffuse sense of weariness, representing a functional state between alertness and sleep (17). From a causal perspective, fatigue is described as a feeling of tiredness and physical discomfort associated with prolonged monotonous work (18). Gander et al. defined fatigue as the inability to perform tasks to established standards due to incomplete recovery from prior work or activities (19). Given its complex psychophysiological nature, the International Maritime Organization (IMO) defines fatigue in its Guidelines on Fatigue as “a reduction in physical and mental capabilities due to physical, mental, or emotional exertion, resulting in diminished strength, speed, reaction time, coordination, or balance” (20). By consolidating these descriptive definitions, Gander et al. argued that “fatigue lacks a universally accepted objective definition but is operationally defined as a decline in perceptual, cognitive, or physiological capacities, leading to reduced ability to perform work safely and effectively” (19).

To take into account the full spectrum and multi-dimensional nature of fatigue as indicated above and in the literature, this review distinguishes four interrelated dimensions of seafarer fatigue. First, sleep-related fatigue or drowsiness refers to fatigue associated with insufficient sleep, poor sleep quality, night work, circadian disruption, and incomplete recovery. Second, cognitive or mental fatigue refers to reduced attention, vigilance, decision-making capacity, and information-processing ability caused by sustained cognitive demands. Third, physical fatigue refers to reduced physical capacity and bodily tiredness associated with manual work, awkward posture, repetitive tasks, or exposure to demanding shipboard environments. Fourth, fatigue-related psychosocial or occupational strain refers to perceived tiredness and exhaustion associated with stress, isolation, family separation, job insecurity, weak organizational support, and poor safety culture. Distinguishing these dimensions is important because different studies may measure different aspects of fatigue, and different types of fatigue may require different prediction and management strategies.

### 1.4 Previous reviews and remaining gaps

Previous reviews have examined determinants of seafarer fatigue, health consequences, and mitigation measures. However, the literature remains fragmented in several respects. First, many studies use the term fatigue to refer to different phenomena, including sleepiness, cognitive fatigue, physical fatigue, and psychosocial strain. Second, seafarers are often treated as a homogeneous group, although fatigue may differ by rank, department, vessel type, voyage pattern, nationality, and organizational context. Third, prediction methods are rarely compared in terms of data requirements, interpretability, external validity, and feasibility for shipboard deployment. Fourth, fatigue risk management strategies are often discussed without clearly distinguishing among empirically supported management pathways, operationally specified but insufficiently validated strategies, and broader conceptual or policy-oriented recommendations.

### 1.5 Aim and review questions

This systematic review and narrative synthesis aims to critically synthesize current evidence on seafarer fatigue, focusing on concepts, risk factors, prediction methods, and risk management strategies. Specifically, this review seeks to clarify how seafarer fatigue has been conceptualized and measured, identify key risk factors under shipboard working conditions, synthesize evidence on subgroup differences among seafarers, compare fatigue prediction methods using a common evaluative framework, and assess the evidence base for fatigue risk management strategies. In doing so, the review aims to provide a more integrated and practice-oriented understanding of seafarer fatigue and to identify priorities for future research and maritime fatigue risk management.

The review is guided by the following questions:

- How has seafarer fatigue been conceptualized and measured in the existing literature?
- What individual, work-related, environmental, sleep-related, organizational, and psychosocial risk factors have been associated with seafarer fatigue?
- How does fatigue differ across seafarer subgroups, such as rank, department, vessel type, voyage pattern, nationality, and organizational context?
- What fatigue prediction methods have been developed, and how do they compare in terms of prediction target, data requirements, interpretability, external validity, and feasibility for shipboard implementation?
- What fatigue risk management strategies have been proposed or evaluated, and how strong is the evidence supporting them, particularly in terms of whether they are empirically supported, operationally specified but insufficiently validated, or mainly conceptual or policy-oriented?

## 2 Methods

To ensure transparency, reproducibility, and systematic handling of the literature, this review followed a structured approach aligned with best practices for systematic and structured reviews.

### 2.1 Search strategy

A comprehensive literature search was conducted across multiple Chinese and English databases, including Web of Science, PubMed, Scopus, China National Knowledge Infrastructure (CNKI), and Wanfang Data. The search period covered January 2000 to April 2026, capturing both foundational studies and recent advances in seafarer fatigue research. A small number of foundational conceptual sources published before 2000 were additionally included through citation searching to support the conceptual framing of fatigue.

Database searches were conducted using a two-level strategy. First, a core search string was constructed by combining maritime population terms with fatigue-related terms. Terms within each block were combined using OR, and the population and fatigue blocks were combined using AND. This core search was designed to identify studies directly related to fatigue among seafarers or maritime personnel. The core English search string was:

(“seafarer*” OR “sailor*” OR “mariner*” OR “ship crew” OR “crew member*” OR “ship operator*” OR “maritime personnel” OR “merchant marine” OR “watchkeeping officer*” OR “officer on watch”)

AND

(“fatigue” OR “sleepiness” OR “drowsiness” OR “tiredness” OR “mental fatigue” OR “physical fatigue” OR “occupational fatigue” OR “sleep quality” OR “circadian rhythm” OR “sleep deprivation” OR “work-rest”)

Second, supplementary topic-specific searches were conducted to ensure coverage of the main review questions. These searches combined the same maritime population and fatigue blocks with one additional thematic block at a time, including prediction methods, risk factors, fatigue risk management, and seafarer subgroup terms. The thematic terms were not combined simultaneously in a single search string, because doing so would require each record to mention all review themes and could exclude relevant studies focusing on only one aspect of seafarer fatigue.

The supplementary thematic terms included the following:

Prediction methods: (“fatigue prediction” OR “fatigue prediction model” OR “Bayesian network” OR “machine learning” OR “deep learning” OR “EEG” OR “physiological monitoring” OR “fuzzy comprehensive evaluation”).

Risk management: (“fatigue risk management” OR “fatigue management” OR “organizational support” OR “safety culture” OR “rest hour compliance” OR “work-rest schedule”).

Influencing factors: (“sleep quality” OR “sleepiness” OR “circadian rhythm” OR “work-related factors” OR “workload” OR “environmental stressors” OR “ship noise” OR “vibration” OR “staffing levels” OR “watchkeeping schedule”).

Seafarer subgroups: (“watchkeeping seafarers” OR “officer fatigue” OR “ratings” OR “deck department” OR “engine department” OR “vessel type” OR “cross-cultural comparison” OR “nationality”).

Equivalent Chinese search terms were used in CNKI and Wanfang Data. Search terms were adapted to the syntax and search functions of each database. Records retrieved from the core and supplementary searches were pooled, duplicates were removed, and the remaining records were screened according to the predefined inclusion and exclusion criteria. No restrictions were placed on study design. Cross-sectional, longitudinal, qualitative, experimental, modeling, review, and official-report studies were considered if they met the predefined inclusion and exclusion criteria.

### 2.2 Inclusion and exclusion criteria

Studies and sources were considered eligible if they met the following criteria. The core evidence base consisted of peer-reviewed journal articles, conference proceedings, and official reports with a primary focus on fatigue among seafarers, ship operators, or maritime personnel. These sources had to be published in either English or Chinese and contain original data, a systematic review, a modeling study, a case analysis, or a theoretical framework relevant to fatigue causes, measurement, prediction, safety outcomes, or management. In addition, a limited number of supplementary conceptual, regulatory, and methodological sources were included when they directly informed the conceptual framework, maritime human-factor context, fatigue prediction methods, or physiological fatigue monitoring relevant to this review. These supplementary sources were used to support interpretation and methodological comparison, rather than as direct evidence of seafarer fatigue prevalence, risk factors, or management effectiveness.

Regarding exclusion criteria, empirical quantitative studies were excluded if they had a sample size of less than 30 or incomplete data, such as missing values exceeding 10% without justification. Duplicate publications were excluded, as were opinion pieces, editorials, or news articles lacking empirical, theoretical, methodological, or regulatory substance. Studies focusing solely on non-maritime populations, such as land-based shift workers, without clear relevance to seafaring or fatigue-related methodological transferability were also excluded. Studies were excluded if they were not published in English or Chinese, or if the full text did not provide sufficient methodological or conceptual information for eligibility assessment and data extraction.

### 2.3 Screening process

The screening process followed three stages:

Title and abstract screening: Initial screening of titles and abstracts was performed to exclude records clearly unrelated to seafarer fatigue. Records were retained for full-text assessment when relevance was uncertain. Two reviewers (ZZ and AQ) checked the screening results, and disagreements or uncertain cases were resolved through discussion with a third reviewer (SH).

Full-text assessment: Full texts of the remaining articles were retrieved and assessed against the inclusion/exclusion criteria. Reasons for exclusion at this stage were documented (e.g., insufficient sample size, missing data, irrelevant outcome).

Final inclusion: Studies passing full-text assessment were included for data extraction and synthesis. The final set included theoretical studies, empirical investigations, and case analyses to ensure both comprehensiveness and scientific rigor.

**Figure 1:**
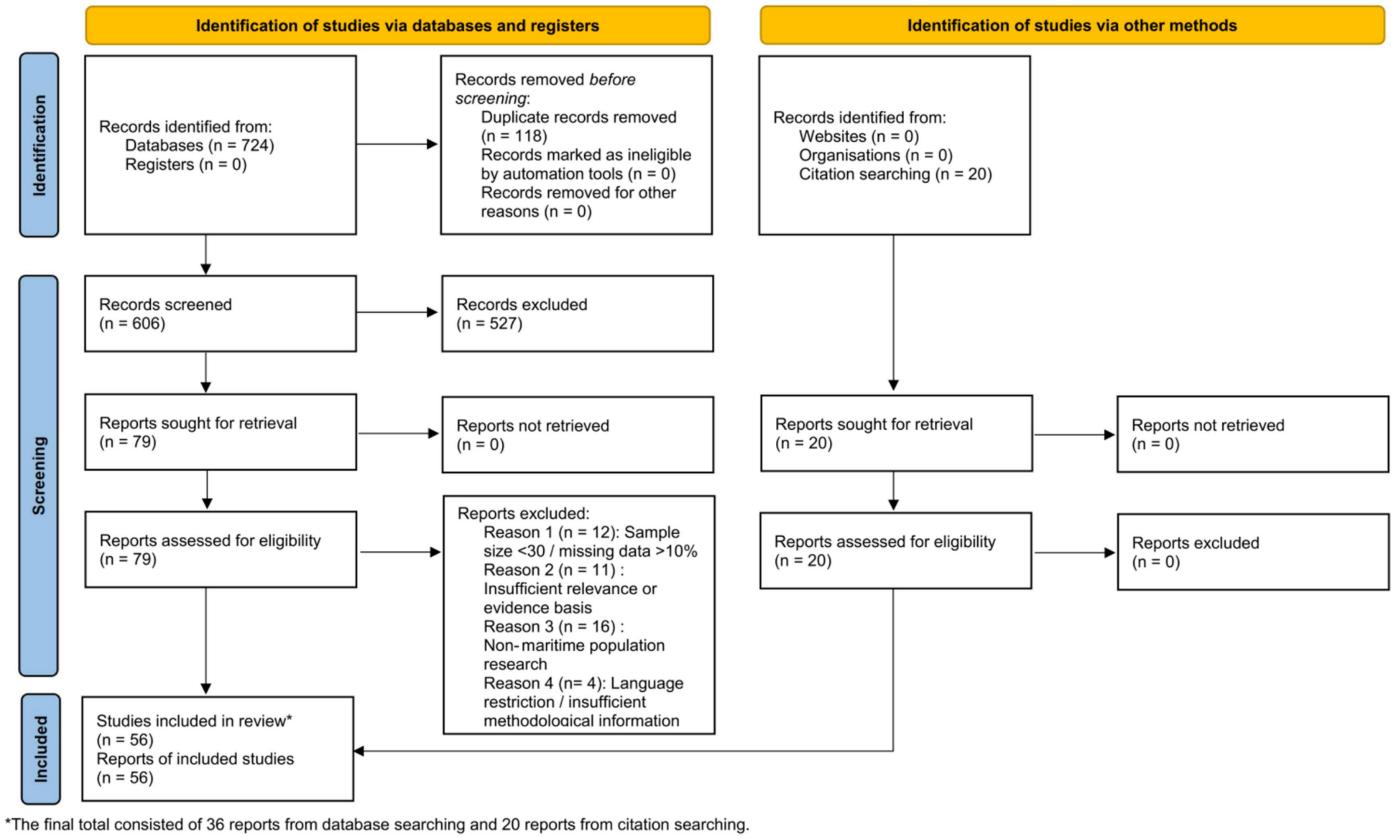
PRISMA 2020 flow diagram of literature search and screening process for seafarer fatigue-related studies (2000–2026).

PRISMA flow diagram: The literature screening process is illustrated in Figure 1.

### 2.4 Quality assessment

The quality of included studies was assessed independently by two reviewers (ZZ and AQ). For cross-sectional survey studies, the assessment was guided by items adapted from the Joanna Briggs Institute (JBI) Critical Appraisal Checklist and the AXIS tool. For qualitative, case-analysis, modeling, review, and official-report studies, appraisal focused on relevance to the review questions, clarity of data sources, transparency of methods, validity of fatigue measurement or interpretation, and practical relevance to maritime settings. Disagreements were resolved through discussion. Studies were not excluded solely on the basis of quality appraisal, but studies with clearer design, larger samples, validated measures, and stronger external validity were given greater interpretive weight in the narrative synthesis.

### 2.5 Data extraction and synthesis

From each included study or source, the following information was extracted: author(s) and year of publication, journal or source, study design or source type, population or operational context, sample characteristics where reported, fatigue focus or measurement approach, main evidence domain, key findings or contributions relevant to the review questions, and major methodological or content limitations.

Synthesis was conducted thematically. Extracted findings were grouped into five major themes corresponding to the structure of this review: concepts and measurement of seafarer fatigue, risk factors and pathways, subgroup differences, fatigue prediction methods, and fatigue risk management strategies. No formal meta-analysis was performed due to heterogeneity in study designs, fatigue definitions, populations, and measurement instruments across the included literature.

### 2.6 Methodological limitations of this review

Several methodological limitations should be acknowledged. First, the inclusion of only English and Chinese language publications may introduce language bias. Second, the heterogeneity of outcome measures precluded meta-analysis. Third, publication bias may exist as studies with significant findings are more likely to be published. Fourth, unpublished studies were not included.

## 3 Results

### 3.1 Overview of the evidence base

The database search identified 724 records. After removal of 118 duplicate records, 606 records were screened by title and abstract. Of these, 527 records were excluded because they were clearly unrelated to seafarer fatigue or did not meet the review scope. The remaining 79 reports were sought for retrieval and assessed for eligibility. Forty-three reports from the database-search branch were excluded at the full-text stage: 12 because of small sample size or missing data exceeding the prespecified threshold, 11 because they lacked original data, review content, or sufficient conceptual, methodological, or regulatory relevance to the review questions, 16 because they focused on non-maritime populations without clear relevance to seafaring, and 4 because of language restriction or insufficient methodological information. Therefore, 36 reports from the database-search branch were included. In addition, citation searching identified 20 further reports, all of which were retrieved, assessed for eligibility, and included. In total, 56 studies were included in the review. In this review, each included study was represented by one report; therefore, the number of included studies and the number of reports were identical.

The included evidence base was methodologically diverse. Most empirical studies used cross-sectional survey designs, commonly relying on self-reported fatigue, sleep quality, perceived workload, stress, or health-related outcomes. A smaller group of studies used interviews, accident cases, simulator experiments, physiological measurements, or fatigue prediction models. This heterogeneity explains why a meta-analysis was not appropriate and why the findings were synthesized narratively rather than statistically. The studies also differed substantially in population, including officers, ratings, cadets, watchkeeping seafarers, fishing crews, tugboat crews, offshore supply crews, and personnel working on cargo, passenger, and roll-on/roll-off vessels.

Table 1 provides an aggregated overview of the evidence base included in this review. Rather than listing all included sources individually in the main text, the table summarizes the reviewed literature by evidence domain, number of references, representative reference numbers, common study designs or data sources, main contribution to the review, and common limitations. Full study/source-level characteristics are provided in Supplementary Table S1.

**Table 1:** Overview of evidence domains included in the review.

Evidence domain	No. of references	Representative references	Typical study designs / data sources	Main contribution to the review	Common limitations
General and conceptual foundations	(4)	(1, 4, 17, 18)	Conceptual articles, longitudinal non-maritime study, review/background sources	Defines general fatigue, shift-work, cognition and stress concepts used to frame seafarer fatigue.	Several sources are conceptual, background, or non-maritime, limiting direct transferability to seafaring contexts.
Seafarer fatigue prevalence, measurement, and risk-factor evidence	(18)	(5, 9, 11, 12, 14, 15, 22, 24, 26, 28, 29, 31)	Field studies, cross-sectional surveys, mixed-methods studies, subjective measurement studies	Provides evidence on fatigue prevalence, sleepiness, work/rest schedules, noise, psychosocial issues and other risk factors.	Often cross-sectional and self-reported; fatigue definitions and measurement tools are inconsistent.
Subgroups, shipboard working conditions, and psychosocial context	(7)	(13, 23, 32–34, 40, 41)	Qualitative studies, case studies, subgroup/working-condition analyses, pandemic/context studies	Shows that fatigue varies by vessel type, department/role, mobility, working conditions and psychosocial context.	Evidence is uneven across subgroups; several studies are context-specific or not primarily fatigue-focused.
Maritime safety, accident analysis, and human reliability	(10)	(2, 3, 8, 10, 27, 30, 37, 46, 48, 53)	Accident-data analysis, Bayesian network, scenario analysis, systematic accident-report review, narrative sources	Links fatigue with maritime accidents, human error, vigilance, safety assessment and accident investigation limitations.	Accident reports may underreport fatigue; models depend on data quality and coding assumptions.
Prediction/evaluation and neurophysiological or AI-based methods	(9)	(7, 21, 44, 45, 47, 49, 54–56)	Machine-learning studies, EEG/neurophysiological studies, simulator experiments, fuzzy/HRA models, method-comparison studies	Supports discussion of objective fatigue monitoring, prediction, model interpretability and feasibility for deployment.	Several studies are non-maritime analogs or simulator/model-based; onboard validation remains limited.
Fatigue management, mitigation, regulation, and compliance	(8)	(6, 16, 19, 20, 25, 36, 51, 52)	Systematic review, guidelines, comparative case studies, mitigation reviews, professional/regulatory discussions	Supports discussion of fatigue-risk management, mitigation measures, company practices, regulation and compliance.	Some sources are policy-oriented or professional discussions rather than tested interventions.

Counts use a primary-category assignment for each included reference. Some references address more than one theme; Supplementary Table S1 records the specific evidence domains and limitations.

The quality appraisal indicated that the strongest evidence generally came from larger cross-sectional studies with clearly defined samples and validated measures, as well as from studies using objective or multi-source data. However, many studies remained limited by self-report bias, cross-sectional design, insufficient control of confounders, limited subgroup analysis, and weak external validation of prediction models. Therefore, evidence was not treated as uniform: studies with clearer design, larger samples, validated instruments, and stronger analytical procedures were given greater interpretive weight in the synthesis.

Overall, the evidence base shows that seafarer fatigue has been studied from several partially overlapping perspectives: occupational health, maritime safety, sleep and circadian disruption, work organization, environmental stressors, human factors, prediction modeling, and fatigue risk management. However, the literature remains fragmented because fatigue is conceptualized and measured differently across studies, subgroup-specific evidence remains limited, and many proposed prediction or management approaches have not yet been validated under real shipboard conditions.

### 3.2 Concepts and measurement of seafarer fatigue

The included studies did not use a single unified definition of fatigue. Instead, fatigue was described as a multidimensional condition involving physical, cognitive, physiological, emotional, and occupational components. Earlier theoretical work distinguished physical fatigue from mental fatigue, while maritime-specific guidelines emphasized the reduction of physical and mental capabilities due to exertion, leading to diminished strength, speed, reaction time, coordination, or balance (17, 20). In this review, seafarer fatigue is therefore understood as a decline in perceptual, cognitive, physiological, or physical capacity that reduces the ability to perform maritime work safely and effectively. As pointed out in the introduction (section 1.3), seafarer fatigue can be distinguished into four interrelated dimensions: sleep-related fatigue or drowsiness, cognitive or mental fatigue, physical fatigue, and psychosocial or occupational strain. This distinction is important because studies measuring “fatigue” may in fact capture different constructs, and different types of fatigue require different prediction and management strategies.

Measurement approaches also varied. The most common approach was self-reported fatigue or sleepiness, including fatigue scales, sleep quality instruments, perceived workload measures, and general health or stress questionnaires. These tools are easy to administer and suitable for large samples, but they are vulnerable to recall bias, under-reporting, social desirability, and differences in cultural interpretation. Other studies measured fatigue indirectly through sleep duration, sleep efficiency, watchkeeping schedule, working hours, or rest-hour compliance. These indicators are useful for evaluating fatigue risk exposure, but they do not always capture actual fatigue because formal rest time may not equal effective recovery.

More recently, Ronca et al. demonstrated the feasibility of neurophysiological assessment in a full-mission bridge simulator by using EEG to assess ship operators’ mental workload, stress, and attention during collision-risk scenarios (21). This supports the relevance of objective human-factor monitoring in maritime safety research. However, direct evidence on continuous physiological monitoring of seafarer fatigue in real shipboard conditions remains limited. Therefore, current measurement practice should be understood as a continuum from subjective self-report, to work-rest exposure indicators, to behavioral and physiological markers. Future studies should combine these sources rather than relying on a single measure.

### 3.3 Risk factors and pathways of seafarer fatigue

The reviewed studies indicate that seafarer fatigue is not a single-factor outcome, but a multidimensional condition produced through several interrelated pathways. Consistent with the conceptual distinction developed in this review, fatigue-related risks can be organized into four major pathways: sleep-related fatigue, cognitive or mental fatigue, physical fatigue, and psychosocial or occupational strain.

#### 3.3.1 Sleep-related fatigue and recovery pathway

Sleep-related fatigue or drowsiness is mainly associated with insufficient sleep, poor sleep quality, fragmented rest, night work, circadian disruption, and incomplete recovery. Several studies showed that short sleep duration and poor sleep quality were strongly related to fatigue among seafarers (11, 14, 15, 22–24). Shipboard conditions may further intensify this pathway. For example, engine noise during sleep can reduce sleep efficiency and increase wake after sleep onset, thereby worsening recovery and contributing to fatigue (15). This finding suggests that formal compliance with rest-hour regulations does not necessarily mean that seafarers obtain effective recovery, because recorded rest time may still be affected by noise, vibration, operational interruptions, or irregular watch schedules.

#### 3.3.2 Cognitive or mental fatigue and workload pathway

Cognitive or mental fatigue is mainly linked to sustained attention, high workload, watchkeeping duties, navigation in complex environments, excessive paperwork, decision-making pressure, and rapid port turnaround (25, 26). Long working hours and high job demands keep seafarers in a prolonged state of mental tension and reduce their capacity for sustained vigilance (27, 28). Working more than 72 h per week has been identified as a direct cause of fatigue among tugboat seafarers (29). Accident-related interviews also showed that insufficient staffing can force seafarers to complete excessive work within a limited time, thereby increasing mental fatigue and reducing operational safety (30). These findings indicate that mental fatigue develops when cognitive demands exceed available recovery resources, especially in safety-critical tasks such as navigation, watchkeeping, cargo operations, and emergency response.

#### 3.3.3 Physical fatigue and shipboard environment pathway

Physical fatigue is associated with manual work, prolonged physical effort, awkward posture, repetitive tasks, vessel motion, poor ergonomic design, and exposure to demanding shipboard environments. Studies have reported that environmental stressors such as noise, vibration, enclosed spaces, heat, and weather conditions can increase bodily strain and fatigue (26, 31, 32). Physical fatigue is therefore not only caused by heavy manual labor, but also by the interaction between work tasks and the physical environment of the ship. Poor workspace design, vibration, and limited accommodation conditions may increase muscular discomfort and reduce the body’s ability to recover after work.

#### 3.3.4 Psychosocial or occupational strain pathway

Psychosocial or occupational strain is related to stress, isolation, work–family conflict, job insecurity, weak organizational support, safety culture, and management practices. Personal and psychosocial factors such as psychological resilience, emotional coping, and family relationships influence how seafarers perceive and tolerate fatigue (29, 31, 33). Work–family conflict was identified as a direct contributor to fatigue among Indonesian tugboat seafarers (29). Organizational and management factors are also important: poor work arrangements, inadequate staffing, weak enforcement of regulations, and insufficient consideration of commuting or non-recorded work time may increase fatigue risk (34–36). This pathway shows that fatigue is not only an individual physiological problem, but also an organizational and social problem shaped by company policies, leadership, manning levels, and reporting culture.

#### 3.3.5 Cumulative and interacting pathways

These four pathways often interact rather than operate independently (37). For example, high workload may shorten sleep time, ship noise may reduce sleep quality, insufficient staffing may increase both physical and cognitive demands, and weak organizational support may reduce fatigue reporting. Wadsworth et al. found that seafarers exposed to five or more fatigue risk factors had a much higher probability of fatigue than those exposed to zero to two risk factors (38). This suggests a cumulative and synergistic mechanism: the more risk pathways that overlap, the greater the likelihood and severity of fatigue.

Overall, the evidence shows that seafarer fatigue should be understood as the result of interacting sleep-related, cognitive, physical, and psychosocial pathways. This pathway-based framework is more consistent with the multidimensional concept of fatigue and provides a clearer basis for subgroup analysis, fatigue prediction, and fatigue risk management.

### 3.4 Subgroup differences in seafarer fatigue

Most studies reviewed seafarers as a relatively homogeneous occupational group, but the available evidence suggests that fatigue differs across subgroups defined by watchkeeping pattern, voyage duration, vessel type, rank, department, nationality, and organizational context. Although subgroup-specific evidence remains limited and sometimes inconsistent, these findings indicate that seafarer fatigue cannot be fully explained by general shipboard exposure alone.

First, watchkeeping pattern appears to influence fatigue through sleep timing and circadian disruption. Oldenburg and Jensen compared sleep quality between watchkeeping at night and day-shift seafarers, finding that watchkeepers had lower sleep efficiency but better sleep quality (39). This finding suggests that different fatigue indicators may not always move in the same direction: objective sleep efficiency, subjective sleep quality, and perceived fatigue may capture different aspects of fatigue experience. Therefore, subgroup comparisons should consider not only fatigue level, but also the measurement method used.

Second, large scale cross-sectional studies focusing on voyage duration revealed less fatigue among long-haul seafarers than in short-haul crews, suggesting an inverse relationship between fatigue and voyage length (12). This is because short-haul operations may involve frequent port calls, rapid turnaround, intensive cargo handling, administrative work, and interrupted rest, which can produce high fatigue even over shorter voyages. Similarly, comparisons between offshore supply vessels and roll-on/roll-off cargo ships showed higher fatigue among crews on roll-on/roll-off cargo ships, which was attributed to frequent port turnovers and multitasking demands, whereas fatigue among offshore supply vessel crews was more closely linked to prolonged sailing time (31). These findings indicate that vessel type should be interpreted together with voyage pattern and operational workload.

Third, nationality and organizational context may shape fatigue exposure and fatigue reporting. Zhao et al. compared fatigue levels between crews of Chinese vessels and ethnically mixed seafarers mustered aboard vessels operated by European shipping companies. It was found that Chinese seafarers across all ranks and departments reported higher levels of fatigue. This was attributed to factors like lower job security, higher work demands, and poorer sleep conditions (6). This suggests that subgroup differences are not only related to biological or occupational characteristics, but also to employment arrangements, organizational support, safety culture, and living conditions on board.

Fourth, evidence on rank and department differences is mixed. Some studies reported that senior officers, including captains, experienced higher fatigue levels because of greater responsibility, higher workload, and decision-making pressure (40–42). However, this pattern was not observed among Indonesian tugboat crews, possibly because smaller crew sizes and more uniform task allocation reduced differences between ranks (29). Similarly, Kim et al. found no significant differences in noise-related sleep disturbance between deck and engine department trainees (43). These inconsistent findings suggest that rank and department alone are insufficient explanations; their effects may depend on vessel size, manning level, task distribution, watch system, and operational context.

Overall, the evidence indicates that subgroup differences in seafarer fatigue exist but remain underexplored. Current studies provide useful comparisons by watchkeeping pattern, voyage duration, vessel type, rank, department, and nationality, but few studies are designed specifically to test subgroup mechanisms. Many subgroup variables are also confounded with each other: for example, vessel type may be linked to voyage pattern, workload, port frequency, and manning level. Therefore, a one-size-fits-all approach to fatigue management is inadequate. Future studies should examine how rank, department, vessel type, voyage pattern, nationality, employment condition, and organizational culture interact to produce different fatigue pathways. Fatigue risk management strategies should also be tailored to these subgroup-specific risk profiles rather than applied uniformly across all seafarers.

### 3.5 Prediction methods for seafarer fatigue

Research on seafarer fatigue prediction can be grouped into three broad categories: empirical formula-based methods, indicator-based evaluation methods, and advanced probabilistic or machine-learning models. These methods differ in their prediction targets, data requirements, interpretability, validation evidence, and practical applicability to shipboard fatigue risk management.

#### 3.5.1 Empirical formula-based methods

Empirical formula-based methods, such as the Ikeda formula and Fatigue Index, represent the simplest approach to fatigue prediction (44). Their prediction target is usually a fatigue score or fatigue risk level derived from limited operational variables, such as working time, rest time, task duration, or duty schedules. The main advantage of these methods is operational feasibility. They require relatively little data, are easy to calculate, and can be used for rapid screening in specific shipboard situations. However, their limitations are also evident. They usually assume a relatively stable relationship between work exposure and fatigue, and therefore have limited capacity to capture individual differences, sleep quality, psychological state, nonlinear interactions, or rapidly changing operational conditions. Their interpretability is high, but their predictive precision and adaptability are relatively weak. Therefore, empirical formulas may be useful for preliminary fatigue risk assessment, but they are insufficient for individualized or dynamic fatigue prediction.

#### 3.5.2 Indicator-based evaluation methods

Indicator-based evaluation methods attempt to assess fatigue by integrating multiple fatigue-related indicators. Zhang et al. improved a fuzzy comprehensive evaluation model by combining Analytic Hierarchy Process and entropy weighting to calculate the relative weights of fatigue indicators, thereby enhancing the objectivity and resolution of fatigue assessment (45). Similar approaches, including Analytic Hierarchy Process and fuzzy comprehensive evaluation, can incorporate work-related, environmental, physiological, psychological, and management-related factors (46, 47). Their prediction target is usually fatigue level assessment or risk grading rather than continuous real-time prediction. Compared with empirical formulas, these models are more comprehensive because they can include multiple dimensions of fatigue. They are also relatively interpretable, which is useful for maritime management and decision-making. However, they still depend heavily on indicator selection and weighting procedures. When weights are based mainly on expert judgment, subjective bias may be introduced; when weights are derived from a specific dataset, their applicability to other vessel types, voyage patterns, and crew groups may be limited. Thus, these methods improve comprehensiveness but still require stronger validation under different shipboard conditions.

#### 3.5.3 Advanced probabilistic and machine-learning models

Advanced probabilistic and machine-learning models represent a more data-driven direction in seafarer fatigue prediction. Bayesian network models are one important example. Russo et al. introduced a Bayesian network model integrating fatigue, stress, and anxiety to estimate human error risks across different ship types, onboard positions, and age groups (48). Strictly speaking, this type of model does not always predict fatigue itself; rather, fatigue is often included as one of several variables used to predict human error or operational risk. Its key advantage is interpretability. Bayesian networks can represent conditional relationships among multiple variables and visualize how fatigue-related factors contribute to risk. They can also be updated when new evidence becomes available. However, their performance depends on the quality of prior knowledge, the reliability of conditional probability estimates, and the empirical validity of the model structure. If the model is mainly expert-driven and lacks external validation, its generalizability to other maritime settings remains uncertain.

Deep-learning models provide another advanced approach. Li and Fu used electroencephalogram signals and a Bidirectional Long Short-Term Memory model to classify seafarer fatigue into three levels, achieving nearly 90% accuracy (49). The prediction target in this case is more direct: fatigue state classification. Compared with empirical formulas and expert-weighted models, deep-learning approaches are better able to process time-series physiological data and capture nonlinear fatigue patterns. This is particularly valuable because fatigue is a dynamic psychophysiological state rather than a fixed outcome. However, these models also have important limitations. High accuracy in a controlled experimental setting does not necessarily guarantee reliable performance in real shipboard environments. Deep-learning models usually require larger datasets, high-quality physiological signals, and careful validation. They may also face problems of overfitting, weak interpretability, sensor noise, motion artifacts, individual variability, and limited crew acceptance. Therefore, their value should be judged not only by accuracy, but also by robustness, transparency, and feasibility for shipboard use (Table 2).

**Table 2:** Comparison of fatigue prediction methods.

Prediction method	Main examples	Prediction target	Data requirements	Interpretability	External validity	Shipboard feasibility
Empirical formula-based methods	Ikeda formula; Fatigue Index	Fatigue score or fatigue risk level	Low; mainly work time, rest time, task duration, or duty schedule data	High	Limited	High
Indicator-based evaluation methods	AHP, entropy weighting, fuzzy comprehensive evaluation	Composite fatigue level or risk grade	Moderate; requires multiple work-related, environmental, physiological, psychological, or management indicators	Moderate to high	Limited to moderate	Moderate
Advanced probabilistic models	Bayesian networks	Fatigue-related human error or operational risk	Moderate to high; requires risk variables, expert knowledge, and conditional probabilities	High	Depends on model validation	Moderate
Machine-learning or physiological models	Deep learning, EEG-based classification, BiLSTM	Fatigue state classification or fatigue onset detection	High; requires large datasets, physiological signals, or time-series data	Low to moderate	Currently limited in real shipboard settings	Limited to moderate

Overall, existing prediction methods show different strengths and limitations. Empirical formulas are simple and easy to implement, but weak in individualization and dynamic prediction. Indicator-based methods are more comprehensive and interpretable, but may depend on subjective weighting and have limited external validation. Bayesian networks offer transparent probabilistic reasoning and are useful for linking fatigue with human error risk, but require reliable model structures and conditional probabilities. Deep-learning and physiological models may improve fatigue-state recognition, but face challenges related to data requirements, interpretability, validation, and real shipboard deployment. At present, no single prediction method can fully adapt to the complex and diverse operational scenarios of maritime navigation. Most existing models have obvious defects such as insufficient anti-interference or poor interpretability in real shipboard application. The evidence instead suggests the need for prediction approaches that balance accuracy, interpretability, data availability, and practical applicability within fatigue risk management systems.

### 3.6 Fatigue risk management strategies

The reviewed literature proposed several fatigue risk management strategies, but their evidence strength varied substantially. To address this issue, the strategies are synthesized according to their evidential status rather than only by the level of responsibility. Three categories were identified: empirically supported management pathways, operationally specified but insufficiently validated strategies, and conceptual or policy-oriented recommendations.

#### 3.6.1 Empirically supported management pathways

The strongest available evidence concerned management pathways associated with fatigue reduction, rather than fully evaluated intervention programs. Kim and Jang, using the Culture-Work-Health Model, found that organizational support could reduce perceived fatigue by enhancing self-efficacy and indirectly improving quality of life (50). This provides empirical support for the role of organizational support, leadership, communication, and psychosocial resources in fatigue management. However, the evidence remains mainly associational. It identifies a plausible pathway through which management practices may reduce fatigue, but it does not demonstrate that a specific shipboard intervention program has been tested and proven effective.

#### 3.6.2 Operationally specified but insufficiently validated strategies

A second group of strategies provided more specific operational responses to different fatigue levels. Li et al. proposed a tiered response plan for ship drivers, in which moderate fatigue could be addressed through short-term alertness-maintaining actions, such as relaxation exercises or chewing actions, whereas severe fatigue would require immediate operational measures, such as arranging support staff or shift rotation (49). This approach is practically useful because it links fatigue level with corresponding management actions. Nevertheless, the available evidence does not yet show that such tiered strategies have been consistently tested across vessel types, voyage patterns, departments, and crewing arrangements. Therefore, these measures should be interpreted as practical operational recommendations rather than established evidence-based interventions.

#### 3.6.3 Conceptual and policy-oriented recommendations

A third group of strategies consisted mainly of conceptual frameworks and policy-oriented recommendations. Wadsworth et al. emphasized that seafarer fatigue results from interacting risk factors and therefore requires a holistic management approach rather than isolated countermeasures (38). Wang similarly proposed a six-dimensional framework with 19 specific measures for preventing seafarer fatigue (36). These frameworks are useful because they reflect the multifactorial nature of fatigue, but they remain broad and mainly conceptual unless their specific components are implemented and evaluated in real shipboard settings.

Policy-oriented recommendations focused on regulatory enforcement, work-rest compliance, and crewing standards. Jepsen et al. argued that effective fatigue management requires stronger legal support and reassessment of regulatory effectiveness and industry compliance, particularly in relation to crew allocation and working conditions (51). Wang further identified loopholes in the implementation of the STCW Convention, including weak oversight by some flag states and insufficient crewing by shipping companies, and proposed raising minimum crewing standards and strengthening Port State Control (52). These recommendations are important because fatigue is partly produced by systemic pressures beyond the control of individual seafarers or single vessels. However, the reviewed evidence provides limited direct evaluation of whether these regulatory measures, as implemented, reduce fatigue at sea.

Overall, the reviewed literature shows an uneven evidence base for fatigue risk management strategies. Organizational support has empirical support as a fatigue-related management pathway, tiered response plans are operationally useful but insufficiently validated, and integrated frameworks or regulatory recommendations remain largely conceptual or policy-oriented. No single strategy was consistently shown to reduce seafarer fatigue across different maritime settings.

## 4 Discussion

### 4.1 Principal findings and contribution of this review

This systematic review and narrative synthesis provides a structured and critical synthesis of current evidence on seafarer fatigue. The main contribution of this review is not simply to restate that fatigue is common among seafarers, but to clarify why the evidence remains fragmented and how it can be organized into a more coherent framework.

First, the review shows that seafarer fatigue is a multidimensional construct. Existing studies often use the same term “fatigue” to describe sleepiness, cognitive or mental fatigue, physical fatigue, and psychosocial or occupational strain. These dimensions overlap, but they do not have identical causes, indicators, or management implications. Sleep-related fatigue is mainly linked to insufficient sleep, poor sleep quality, night work, circadian disruption, and incomplete recovery. Cognitive fatigue is more closely related to reduced vigilance, attention, decision-making, and situational awareness during safety-critical tasks. Physical fatigue reflects bodily tiredness and reduced physical capacity under demanding shipboard conditions. Psychosocial strain is associated with stress, isolation, job insecurity, weak organizational support, and poor safety culture.

Second, the findings suggest that fatigue should be understood as a cumulative pathway rather than as the result of isolated risk factors. Shipboard fatigue emerges from the interaction between work organization, manning level, watchkeeping schedules, port turnaround, environmental stressors, sleep opportunity, individual characteristics, and safety culture. Therefore, formal compliance with work-rest hour rules does not necessarily mean effective recovery if rest is fragmented, interrupted, or taken under poor sleeping conditions.

Third, this review highlights the heterogeneity of seafarers. Fatigue may differ by rank, department, vessel type, voyage pattern, nationality, and organizational context. However, the evidence remains limited and sometimes inconsistent. For example, short-sea operations may increase fatigue through frequent port calls and interrupted rest, whereas long voyages may produce cumulative fatigue through prolonged monotony and extended time at sea. Similarly, vessel type, department, rank, workload, and crewing arrangements are often intertwined. This means that one-size-fits-all fatigue management is unlikely to be effective.

The apparently inconsistent findings across studies should therefore not be interpreted simply as contradictory evidence. Rather, they reflect differences in what is being measured, who is being studied, and under what operational conditions fatigue occurs. For example, studies using sleep efficiency, self-reported sleep quality, perceived fatigue, or accident-related indicators may capture different aspects of the fatigue process. Similarly, findings on voyage duration, rank, department, or vessel type may vary because these variables are often confounded with port frequency, workload intensity, manning level, watchkeeping system, and organizational support. This suggests that seafarer fatigue is better understood as a context-dependent and pathway-specific phenomenon than as a uniform occupational outcome.

Compared with previous reviews, this review contributes by integrating four issues that are often treated separately: conceptual heterogeneity, risk pathways, subgroup differences, and the evidence strength of prediction and management approaches. It therefore responds to the need for a more critical and practice-oriented synthesis of seafarer fatigue research.

### 4.2 Implications for maritime safety and accident prevention

The findings confirm that fatigue is not only an occupational health problem, but also a maritime safety issue. Fatigue can impair vigilance, reaction time, judgment, communication, and situational awareness, thereby increasing the likelihood of navigation errors, collisions, groundings, and other incidents. However, recent evidence also suggests that fatigue may be under-identified in official accident investigations. For example, a recent systematic review of marine casualty reports found that fatigue was cited in only 29 cases, or 5.6%, despite its widely recognized role in maritime human error. This suggests that fatigue may remain a latent and underreported risk factor in maritime safety systems (53).

This has two implications. First, accident investigation should pay more attention to fatigue-related information, including recent sleep history, work-rest records, watch schedules, workload, environmental conditions, and organizational pressures. Second, fatigue should be treated as a system-level safety issue rather than as an individual failure. If fatigue is produced by insufficient manning, unrealistic schedules, frequent interruptions, poor accommodation conditions, or weak reporting culture, then responsibility cannot be placed only on individual seafarers.

A key practical problem is that fatigue is often less visible than technical failure or immediate operator error. Accident investigations may rely on formal rest-hour records, but these records do not necessarily show whether rest was uninterrupted, whether sleep was restorative, or whether the crew member was affected by cumulative workload, night work, port turnaround, or psychosocial strain. Therefore, accident investigation procedures should move beyond checking formal compliance and include fatigue-sensitive questions on sleep opportunity, sleep quality, recent workload, operational interruptions, and organizational pressures before the incident.

### 4.3 Implications for fatigue prediction

The reviewed prediction methods differ substantially in their practical value. Empirical formula-based methods are simple, interpretable, and easy to apply, but they are weak in individualization and dynamic prediction. Indicator-based evaluation methods can integrate work-related, environmental, physiological, psychological, and management indicators, but their validity depends heavily on indicator selection, weighting procedures, and validation across different shipboard contexts. Bayesian networks and machine-learning models can capture probabilistic and nonlinear relationships, but they require reliable data, careful model validation, and sufficient interpretability.

Recent neurophysiological studies provide useful methodological insights. In the maritime domain, Ronca et al. showed that EEG can be used in a full-mission bridge simulator to assess ship operators’ workload, stress, and attention during collision-risk scenarios (21). Driving studies have further shown that EEG-based fatigue labeling may detect fatigue onset more precisely than simple time-on-task assumptions (54, 55), and that the MDrow index may be sensitive to both passive and active mental fatigue (56). However, these findings should be interpreted cautiously. Most of this evidence comes from simulator or non-maritime driving settings, so it cannot yet be treated as direct evidence for routine shipboard fatigue monitoring. Future maritime prediction models should therefore be evaluated not only by accuracy, but also by external validity, interpretability, sensor burden, crew acceptance, privacy, and feasibility for real shipboard deployment.

For real-world maritime operations, AI- or machine-learning-based fatigue prediction should be implemented as decision-support rather than as an automatic decision-making tool. At the current stage, such models may be most useful for risk screening, early warning, and identifying high-risk combinations of workload, watchkeeping schedule, sleep disruption, and environmental stressors. Their outputs should be interpreted together with seafarer self-reports, supervisor observations, work-rest records, and operational context. Before onboard deployment, prediction models should be externally validated across different vessel types, voyage patterns, departments, nationalities, and crewing arrangements. They should also be designed to minimize sensor burden, protect crew privacy, and avoid punitive use of fatigue data; otherwise, crew acceptance and reporting culture may be weakened.

### 4.4 Implications for fatigue risk management

The evidence base for fatigue risk management is uneven. Some management pathways have empirical support, especially those related to organizational support, leadership, communication, sleep opportunity, and workload reduction. These findings suggest that fatigue can be reduced when companies and shipboard leaders provide better support, protect rest opportunities, and recognize fatigue as a legitimate safety risk.

However, many proposed strategies remain insufficiently validated. Tiered response plans, such as assigning support staff, adjusting shifts, or using short-term alertness-maintaining actions, are operationally useful, but they have not been consistently tested across vessel types, voyage patterns, departments, and crewing systems. Broader frameworks and regulatory recommendations, including improved enforcement of work-rest rules, stronger crewing standards, and integrated fatigue risk management systems, are important but remain largely conceptual or policy-oriented unless their actual effects are evaluated in real maritime settings.

Therefore, the review suggests a layered approach to fatigue risk management. At the regulatory and company level, work-rest compliance should be combined with adequate manning, realistic scheduling, and stronger monitoring of actual recovery. At the shipboard level, fatigue management should protect sleep, reduce unnecessary interruptions, redistribute workload during high-demand periods, and improve accommodation conditions such as noise, vibration, heat, and lighting. At the safety-culture level, fatigue reporting should be non-punitive so that crew members can report fatigue without fear of blame or job insecurity.

Building on this layered management framework and the limitations of existing AI and neurophysiological fatigue prediction models discussed earlier, we put forward targeted, operable implementation guidelines for intelligent fatigue monitoring technologies in real maritime scenarios.

First, a phased promotion mode is recommended for different vessel types. For ocean-going vessels and passenger ships with stable navigation and sufficient crew, portable EEG and fatigue monitoring devices can be piloted to track on-duty officers’ status. For short-sea vessels and tugboats suffering from severe environmental interference, simple indicator evaluation and empirical formulas are preferred to avoid false alarms.

Second, standardized usage rules should be formulated for onboard monitoring equipment. Sensor installation needs to adapt to ship vibration and crew activities to reduce signal artifacts. Devices are suggested to operate only during high-risk watch periods, and relevant physiological data must be protected in compliance with laws and maritime regulations, to raise crew acceptance.

Third, a dual supervision mechanism combining intelligent alerts and manual management shall be established. Mild fatigue warnings correspond to temporary work adjustments, while severe alarms require immediate shift rotation and complete record-keeping.

Fourth, shipping enterprises and research institutions can cooperate to accumulate real onboard data, which helps optimize model performance and improve the anti-interference capability of prediction tools for practical maritime application.

### 4.5 Research gaps and future directions

Several research gaps remain. First, subgroup-specific evidence should be strengthened. Future studies should move beyond treating seafarers as a homogeneous group and examine how fatigue differs by rank, department, vessel type, voyage pattern, nationality, employment conditions, and company culture. Such work should not only compare fatigue levels across groups, but also explain the mechanisms through which different risk factors produce different fatigue experiences.

Second, future research should develop pathway-based and multifactorial models of fatigue. Shipboard fatigue is shaped by the interaction of work organization, sleep and recovery, environmental stressors, individual characteristics, and safety culture. Therefore, future studies should examine nonlinear relationships, cumulative effects, and interactions among these factors rather than testing isolated predictors.

Third, fatigue prediction models require stronger external validation and practical evaluation. Hybrid approaches that combine interpretable statistical or Bayesian methods with machine-learning techniques may help capture nonlinear patterns and individual variability while maintaining explainability. However, such models should be assessed not only by predictive accuracy, but also by data requirements, interpretability, robustness, crew acceptance, and feasibility for shipboard deployment.

Fourth, fatigue management strategies should become more context-specific and evidence-based. Future studies should evaluate whether interventions such as watch schedule redesign, workload redistribution, sleep protection, environmental improvement, and fatigue reporting systems actually reduce fatigue under real shipboard conditions. Management protocols should be tailored to vessel type, voyage duration, operational workload, and crew characteristics rather than applied as generic recommendations.

## 5 Conclusion

This review shows that seafarer fatigue is a multidimensional and system-level problem shaped by sleep and recovery, work organization, shipboard environment, individual characteristics, organizational support, and safety culture. Although existing studies have improved understanding of fatigue prevalence, risk factors, prediction methods, and management strategies, the evidence remains fragmented and uneven. Stronger evidence is available for the role of sleep disruption, demanding work schedules, environmental stressors, and organizational conditions, whereas subgroup-specific mechanisms, prediction models, and management interventions still require stronger validation in real shipboard settings. Future research should adopt clearer fatigue concepts, stronger subgroup analysis, externally validated prediction methods, and evidence-based fatigue risk management strategies. In practice, reducing seafarer fatigue requires integrated approaches that combine adequate crewing, protected rest, safer work organization, supportive safety culture, and validated monitoring tools. In terms of emerging artificial intelligence and electroencephalography-based fatigue monitoring technologies, the maritime industry should adopt a rational application strategy. Given the obvious gaps between simulator experiments and real onboard operating environments, relevant technologies shall be promoted by category and in phases. Matched vessel management systems and data protection regulations are also indispensable. Integrating advanced monitoring technologies with systematic maritime management can effectively translate theoretical research into practical safeguards for seafarers’ well and navigation safety.
